# Caught up by Geopolitics: Sanctions and the EU’s Response to Russia’s War Against Ukraine

**DOI:** 10.1515/ev-2023-0051

**Published:** 2023-10-12

**Authors:** Katharina Meissner

**Affiliations:** Department of Political Science, EIF, University of Vienna, Wien, 1010, Austria

**Keywords:** European Union, geopolitics, sanctions, Russia, F13, F51

## Abstract

Russia’s war against Ukraine unfolded unprecedented geopolitical, economic and energy-related tension in the European Union (EU). Amidst this situation, decision-makers and scholars alike have returned to concepts of geopolitics to reflect the EU’s external affairs and its position in the international (trade) system. Indeed, geopolitical considerations help us to understand the sanctions imposed by the EU against Russia. The restrictive measures against Russia and the Russian-controlled areas in Ukraine are, to date, the most comprehensive measures adopted autonomously by the EU. They reflect the EU’s geopolitical considerations in carving out a strong response to Russia’s military aggression against Ukraine and the unparalleled political and security threat in Europe.

## 1 Introduction: A Return of Geopolitics?

For more than 10 years now, the European Union (EU) and its member states have been under considerable distress and have faced multiple crises (Jones, Kelemen, and Meunier 2021; Zeitlin, Nicoli, and Laffan 2019). Russia’s war on Ukraine added unprecedented economic, energy-related and geopolitical tension to this situation and unsettled Europe’s security architecture.

Russia’s military aggression against Ukraine catapulted decision-makers and scholars into a massively changed system of international relations and required a new perspective on international trade and global politics. While for a long time European policy-makers hailed the liberal world order and economic interdependence, recent crises and, at the latest, Russia’s war against Ukraine unleashed a return of power politics, economic protectionism, and geopolitics.

Indeed, against the background of these massively changed dynamics in global trade and international relations, scholars have returned to concepts of geopolitics and geoeconomics to analyze current international politics. The EU and its external affairs, too, are increasingly scrutinized through such a lens (e.g. Meunier and Nicolaidis 2019; Moraes and Wigell 2022; Weinhardt et al. 2022). This is a remarkable shift in scholarly perspective on the EU in global affairs. Until recently, power politics and a strategic use of economic power – key components of geopolitics and geoeconomics – were hardly ascribed to the EU (e.g. Hyde-Price 2006; Meissner 2018; Zimmermann 2007). Hence, the current shift in attention paid to geopolitics and geoeconomics is a much-needed reality check in the literature.

## 2 Geopolitical Considerations in Sanctions Decisions

Geopolitical and geoeconomic thinking rests on an understanding of world politics in which states and state-like-entities like the EU compete for power. Geopolitics is not necessarily limited to territorial, geographical aspects of power. Rather, in today’s world and especially for the EU, power is inherently linked to economics, to the EU’s position in the global market, and its ability to reduce one-sided dependencies on other countries (e.g. Aggarwal and Reddie 2020; Meissner 2018). Geoeconomics provides an even more differentiated perspective on the EU’s external relations and its position in the international (trade) system. The concept refers to the ‘geostrategic use of economic power’ (Wigell 2016, p. 137) in the service of political and security ambitions. Geoeconomics, thus, grasps economic aspects of strategic political and security competition among states and state-like entities (Aggarwal and Reddie 2020).

Recently, we observe a shift in scholarly perspective on the EU in global affairs and international trade. Increasing attention is paid to geostrategic considerations in analyzing EU external relations and the respective policy decisions (e.g. Meissner 2023; Meissner and Graziani 2023; Nitoiu and Sus 2019). This shift is also evident in the literature on sanctions and studies on how policy-makers adopt and design sanctions packages (Hedberg 2018; Meissner 2023; Meissner and Graziani 2023). Hedberg (2018), for instance, proposes a perspective on the design of Russian countersanctions against Europe which she labels ‘differentiated retaliation’. Differentiated retaliation considers geostrategic concerns when decision-makers adopt sanctions. They do so in such a way that they choose a design which hurts the targeted states most (Hedberg 2018). This perspective puts forward a strategic alignment of economic, security, and political activities in the area of sanctions.

From a geopolitical perspective, sanctions can be understood as a tool to promote foreign policy and security goals through economic means – they are a form of economic statecraft. Scholarship finds in recent studies that even the EU increasingly makes use of sanctions as economic statecraft (Meissner 2023; Meissner and Graziani 2023) as will also be shown in this article. Under the condition of geopolitical concerns, we would expect decision-makers to opt for large scale sanctions which impose high costs on the target state (e.g. Giumelli 2011). The underlying assumption is that sanctions shall be an appropriate and proportionate response to the misbehavior of the target state (Giumelli 2011). Thus, when activities of the target state pose a geopolitical or security threat to the international community, we can expect policy-makers to maximize the costs imposed by sanctions for that very state. Large scale, comprehensive sanctions arguably exert high pressure on the target state and incur heavy costs. Compared to smart, targeted sanctions they discriminate the least among the population of the target state and, thus, come with high costs for the country, its sectors and population (Biersteker et al. 2013; Meissner 2023; Meissner and Graziani 2023).

## 3 Comprehensive Sanctions Against Russia

It is the EU’s principled position to adopt smart, targeted sanctions. They shall avoid unintended, humanitarian consequences for the population in the target state. Such unintended, negative externalities of sanctions and their potential effects detrimental to the intended objectives are well documented in the literature (e.g. Eichenberger and Stadelmann 2023; Reza Farzanegan and Batmanghelidj 2023). Yet, it is evident that sanctions against Russia are the most comprehensive ever imposed autonomously by the EU. The large-scale restrictive measures imposed by the EU and their comprehensive design can be understood as a reaction to the unprecedented geopolitical and security threat by Russia. They reflect the geopolitical considerations of EU policy-makers in reaction to Russia’s military aggression and unparalleled security threat as will be argued here.

Indeed, sanctions imposed by the EU shall pursue their objectives effectively and shall be designed in such a way that they minimize unintended, negative externalities for the population in the target state. The vast majority of EU restrictive measures are smart sanctions and target specific individuals, products and entities (Giumelli, Hoffmann, and Książczaková 2020). In the context of EU sanctions against Russia, the European Commission insists on their targeted nature and on the commitment to avoiding unintended consequences for the civilian population (European Commission 2022a). For this reason, the sanctions foresee manifold exceptions for humanitarian and medical purposes, the satisfaction of food security and basic needs (Meissner and Graziani 2023).

**Figure 1:**
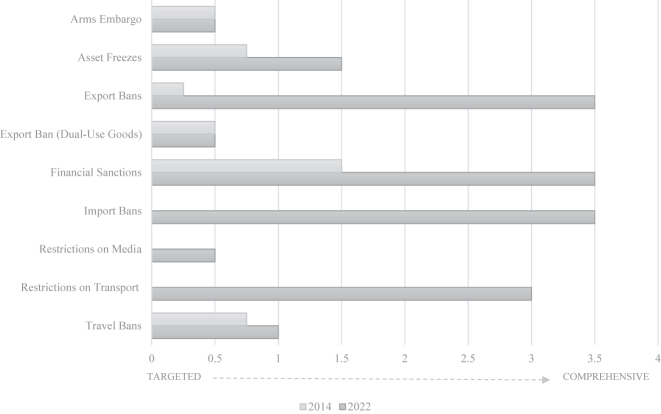
Scope of EU sanctions against Russia (2014–2022). Source: Meissner and Graziani (2023, p. 383, Figure 1).

However, the gravity of Russia’s unprovoked military aggression of Ukraine resulted in the adoption of sanctions of unprecedented breadth and depth. Over time since 2014 and especially since February 2022 when Russia invaded Ukraine, the number and the scope of the restrictive measures in place against Russia and the Russian-controlled areas in Ukraine massively expanded and shifted from targeted towards comprehensive (Figure 1; Meissner and Graziani 2023). The sanctions’ increasing scope and depth have also become costlier for the Russian civilian population with restrictions in the economic, financial and transport sectors that affect the public’s every-day life.

A recently published dataset on the transformation and design of EU restrictive measures against Russia maps the scope and design of the entire set of sanctions imposed against Russia (Meissner and Graziani 2023): by early 2023, the EU has imposed travel bans on 967 individuals as well as asset freezes on 967 natural persons and on 50 legal persons, including several Russian banks and financial institutions, companies engaged in various industries, including the arms, petroleum, electronics and transport industry as well as on legal persons supporting and/or benefitting from the Russian government. The export, import and related services bans were extended to multiple core economic sectors: the military, defense and security sector; the oil and gas sector; the aviation and space industry; the maritime industry; and the luxury industry. Financial restrictions were massively expanded in their range and scope: they were extended to a wide range of financial institutions, including the Central Bank of Russia and Russian Direct Investment Fund. In addition, multiple Russian banks were excluded from the SWIFT system. These observations indicate clearly that overall the scope of EU sanctions against Russia has become more comprehensive over time.

## 4 Maximizing Costs for Russia and the Russian Economy

The unprecedented comprehensive design of sanctions against Russia suggests that the EU, out of geopolitical considerations, seeks to maximize the costs for Russia and the Russian economy. Indeed, the aim of the restrictive measures is to significantly weaken the Russian economy in order to ‘cripple the Kremlin’s ability to finance the war’ (European Commission 2022b). Russia’s unprovoked military aggression of Ukraine is seen as an unprecedented threat to the international community and Europe, and the war’s geopolitical implications are a source of major concern for the EU (EEAS 2022).

The EU is outspoken about the fact that it seeks to inflict the highest possible economic and political costs on Russia as a response to this significant threat (European Commission 2022b). This was made clear by European Commission President Ursula von der Leyen when she described the restrictive measures as the ‘toughest sanctions the world has ever seen’ which shall contribute to the outcome that ‘Russia’s financial sector is on life-support’ and ‘Russia’s industry is in tatters’ (European Commission 2022c). Compared to previous restrictive measures, the EU’s sanctions against Russia are designed in such a way as to maximize the costs for the target state and to be punitive in nature (see also Moret 2022).

Hence, in the case of Russia, the restrictive measures are an economic means used by the EU to constrain the Russian government in its ability to further pursue the war against Ukraine. The sanctions align an economic tool with foreign policy goals in the service of EU geostrategic ambitions.

## 5 The EU as a Geopolitical Actor

Sanctions are not the only policy instrument through which the EU pursues geostrategic ambitions. Indeed, the EU started to conceptualize and position itself as a geopolitical and geoeconomic actor. With the incoming Commission President Ursula von der Leyen in 2019 came an assertive, ‘geopolitical’ European Commission. The self-declared ‘geopolitical’ European Commission can be understood as a response to the massively shifted international relations and the global disorder of recent years (Lavery and Schmidt 2021). The European Commission’s notion of geopolitics rests on an assertive promotion of the EU’s economic, regulatory and political power by aligning economic and foreign policy in a more strategic fashion.

The EU’s notion of geopolitics and its ambition to align economic and foreign policy in a more strategic way finds expression in a number of recent strategies by the European Commission and a range of policy measures. According to the latest trade strategy, the Trade Policy Review (European Commission 2021), for example, trade policy shall be aligned with the EU’s geopolitical interests and the Open Strategic Autonomy – a stronger focus on the EU’s own interests and preferences and its resilience in a massively changed international (trade) system. The European Economic Security Strategy (European Commission and High Representative 2023) is arguably the most strategic document to date when it comes to an alignment of economic and foreign policy. The strategy aims at explicitly aligning trade policy and trade instruments with matters of the CFSP.

As the EU revised its trade strategy and its position as a geopolitical actor, it also devised a range of new (trade) policy instrument and adapted some of its established policy instruments in order to provide a more effective response to a changed international trade system. These instruments seek to tackle economic distortions by third countries, defend the EU and its member states against economic coercion, link political goals such as normative values and sustainability to the EU’s actions, and to ensure Europe’s critical infrastructure and supply resilience (Gehrke 2022). They are offensive and defensive market-directing measures to ensure the EU’s economic and political interests and to position the EU as a resilient and strong actor in global affairs. What makes them different from previous trade strategies and policy instruments is their focus on market-direction and protection of Europe’s economy in alignment with political and strategic ambitions. Hence, the EU increasingly designs economic measures in the service of political and security objectives in a strategic fashion.

## 6 Conclusions

Russia’s invasion of Ukraine on 24 February 2022, marked an unparalleled threat to the European and international order. The EU’s response to Russia’s aggression against Ukraine resulted in the by far most comprehensive sanctions it has ever adopted autonomously to date. The large-scale sanctions were carved out as a geopolitical response to Russia’s military and security threat in Europe.

A recently published dataset (Meissner and Graziani 2023) documents the entire set of EU restrictive measures imposed on Russia and the Russian-controlled areas in Ukraine since 2014. The data show the significant transformation of these sanctions from a rather targeted to an unprecedented comprehensive design in 2023. The EU’s principled position is to avoid humanitarian consequences for the sanctioned targets’ broader population and, indeed, this is reflected in the humanitarian exemptions introduced in the set of sanctions against Russia and the Russian-controlled areas in Ukraine. Yet, the priority of humanitarian considerations seems to have shifted in 2022 and 2023 following the unprecedented threat imposed by Russia in favor of a strong reaction from the EU.

Sanctions against Russia are an economic means to inflict major costs on the Russian economy and to constrain its ability to pursue the war against Ukraine. They are an economic means by the EU to pursue political and security objectives in a strategic fashion. Restrictive measures are not the only policy tools the EU uses to promote its political and strategic ambitions. Rather, the more assertive use of sanctions can be understood as a revised strategic positioning of the EU as a ‘geopolitical’ actor. By geopolitics, the EU means a stronger promotion of European interests and preferences through an alignment of economic and foreign policy tools in a strategic fashion. We observe such an alignment in the use of restrictive measures, but also in the EU’s trade and industrial policies and the adoption of newly design policy instruments. The EU has arguably started to position itself in new, geopolitical age.

## References

[j_ev-2023-0051_ref_001] Aggarwal Vinod K., Reddie Andrew W. (2020). New Economic Statecraft: Industrial Policy in an Era of Strategic Competition. *Issues & Studies: A Social Science Quarterly on China, Taiwan and East Asian Affairs*.

[j_ev-2023-0051_ref_002] Biersteker T., Eckert S. E., Tourinho M., Hudáková Z. (2013). *Effectiveness of UN Targeted Sanctions: Findings from the Targeted Sanctions Consortium (TSC)*.

[j_ev-2023-0051_ref_003] EEAS (2022). *Taking Action on the Geopolitical Consequences of Russia’s War*.

[j_ev-2023-0051_ref_004] Eichenberger Reiner, Stadelmann David (2023). Sanctions Are Costly for Citizens but Beneficial for Autocrats: A Political-Economic Perspective. *The Economists’ Voice*.

[j_ev-2023-0051_ref_005] European Commission (2021). Communication from the Commission to the European Parliament, the Council, the European Economic and Social Committee and the Committee of the Regions: Trade Policy Review – An Open, Sustainable and Assertive Trade Policy.

[j_ev-2023-0051_ref_006] European Commission (2022a). *Commission Guidance Note on the Provision of Humanitarian Aid in Compliance with EU Restrictive Measures (Sanctions)*.

[j_ev-2023-0051_ref_007] European Commission (2022b). *EU Sanctions Against Russia Following the Invasion of Ukraine*.

[j_ev-2023-0051_ref_008] European Commission (2022c). *State of the Union Address by President von der Leyen*.

[j_ev-2023-0051_ref_009] European Commission and High Representative (2023). *An EU Approach to Enhance Economic Security*.

[j_ev-2023-0051_ref_010] Gehrke Tobias. (2022). EU Open Strategic Autonomy and the Trappings of Geoeconomics. *European Foreign Affairs Review*.

[j_ev-2023-0051_ref_011] Giumelli F., Hoffmann F., Książczaková A. (2020). The when, what, where and Why of European Sanctions. *European Security*.

[j_ev-2023-0051_ref_012] Giumelli Francesco. (2011). *Coercing, Constraining and Signalling: Explaining Un and Eu Sanctions After the Cold War*.

[j_ev-2023-0051_ref_013] Hedberg Masha. (2018). The Target Strikes Back: Explaining Countersanctions and Russia’s Strategy of Differentiated Retaliation. *Post-Soviet Affairs*.

[j_ev-2023-0051_ref_014] Hyde-Price Adrian. (2006). ‘Normative’ Power Europe: A Realist Critique. *Journal of European Public Policy*.

[j_ev-2023-0051_ref_015] Jones Erik, Kelemen Daniel R., Meunier Sophie (2021). Failing Forward? Crises and Patterns of European Integration. *Journal of European Public Policy*.

[j_ev-2023-0051_ref_016] Lavery Scott, Schmid Davide (2021). European Integration and the New Global Disorder. *Journal of Common Market Studies*.

[j_ev-2023-0051_ref_017] Meissner Katharina L. (2018). *Commercial Realism and EU Trade Policy: Competing for Economic Power in Asia and the Americas*.

[j_ev-2023-0051_ref_018] Meissner Katharina L. (2023). How to Sanction International Wrongdoing? the Design of EU Restrictive Measures. *Review of International Organizations*.

[j_ev-2023-0051_ref_019] Meissner Katharina L., Graziani Chiara (2023). The Transformation and Design of EU Restrictive Measures against Russia. *Journal of European Integration*.

[j_ev-2023-0051_ref_020] Meunier Sophie, Nicolaidis Kalypso (2019). The Geopoliticization of European Trade and Investment Policy. *Journal of Common Market Studies*.

[j_ev-2023-0051_ref_021] Moraes Henrique Choer, Wigell Mikael, Babić Milan, Dixon Adam D., Li Imogen T. (2022). Balancing Dependence: The Quest for Autonomy and the Rise of Corporate Geoeconomics. *The Political Economy of Geoeconomics: Europe in a Changing Word*.

[j_ev-2023-0051_ref_022] Moret E. (2022). Sanctions and the Costs of Russia’s War in Ukraine. ..

[j_ev-2023-0051_ref_023] Nitoiu Christian, Sus Monika (2019). Introduction: The Rise of Geopolitics in the EU’s Approach in its Eastern Neighbourhood. *Geopolitics*.

[j_ev-2023-0051_ref_024] Reza Farzanegan Mohammad, Batmanghelidj Esfandyar (2023). Understanding Economic Sanctions on Iran: A Survey. *The Economists’ Voice*.

[j_ev-2023-0051_ref_025] Weinhardt Clara, Mau Karsten, Pohl  Jens Hillebrand, Babić Milan, Dixon  Adam D., Liu Imogen T. (2022). The EU as a Geoeconomic Actor? A Review of Recent European Trade and Investment Policies. *The Political Economy of Geoeconomics: Europe in a Changing Word*.

[j_ev-2023-0051_ref_026] Wigell Mikael. (2016). Conceptualizing Regional Powers’ Geoeconomic Strategies: Neo-Imperialism, Neo-Mercantilism, Hegemony, and Liberal Institutionalism. *Asia Europe Journal*.

[j_ev-2023-0051_ref_027] Zeitlin Jonatha, Nicoli Francesco, Laffan Brigid (2019). Introduction: The European Union beyond the Polycrisis? Integration and Politicization in an Age of Shifting Cleavages. *Journal of European Public Policy*.

[j_ev-2023-0051_ref_028] Zimmermann Hubert. (2007). Realist Power Europe? the EU in the Negotiations about China’s and Russia’s WTO Accession. *Journal of Common Market Studies*.

